# Supplementary material for: Risk factors for sacrococcygeal pilonidal sinus: a systematic review and meta-analysis supplemented by genetic causal assessment
Source: Front Surg. 2026 Jan 7;12:1718589. doi: 10.3389/fsurg.2025.1718589 (PMC12819706; doi:10.3389/fsurg.2025.1718589)
Supplement: Supplementary file 2 [file Datasheet2.zip › Supplementary Data 2/MR_pipeline_p5e-6/funnel_plots/ukb-ppp-COL3A1-OID30379.finngen_R12_L12_PILONIDALCYST.pdf]

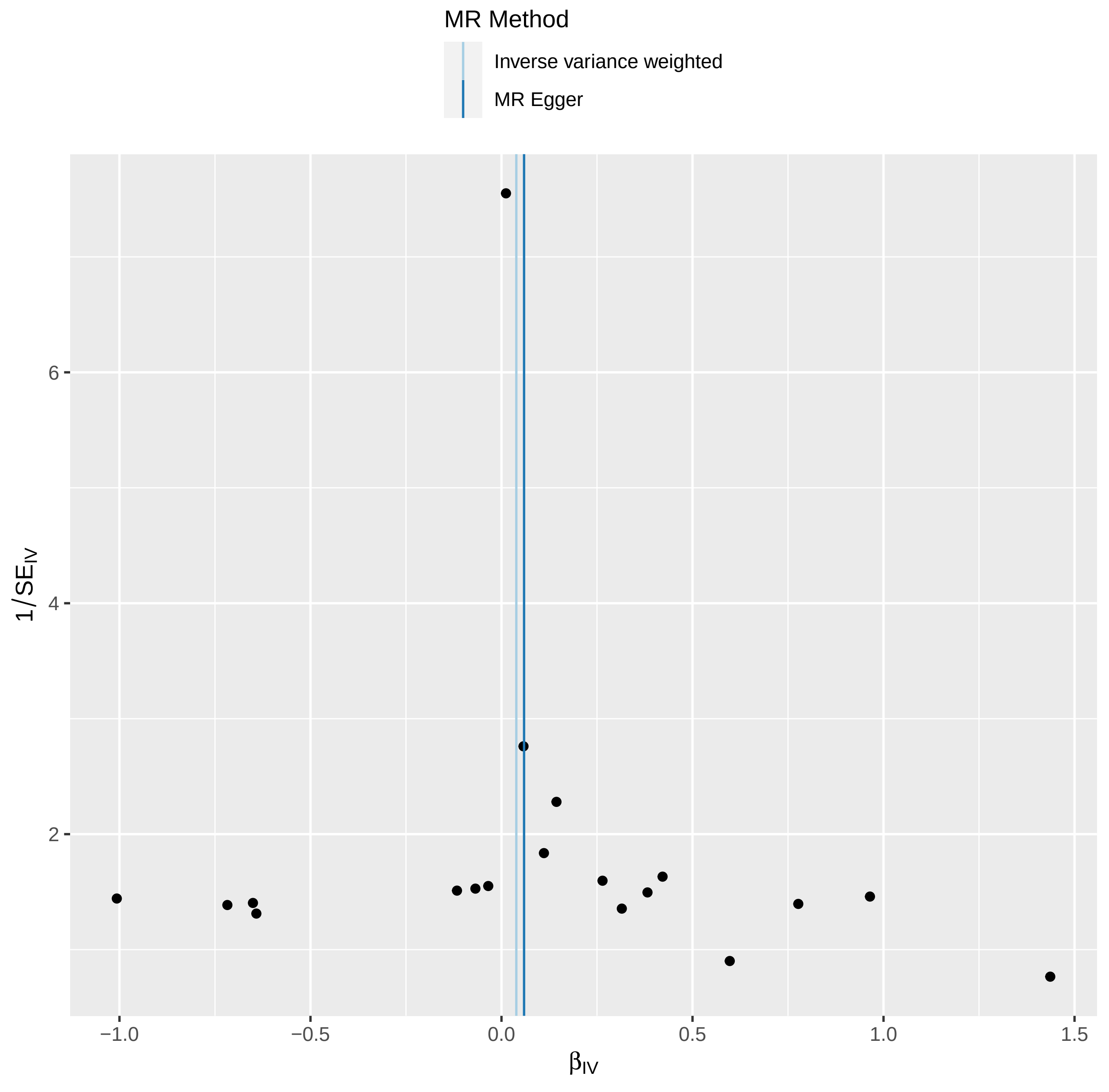
MR Method
Inverse variance weighted
MR Egger
6
4
2
$1/SE_{IV}$
−1.0
−0.5
0.0
0.5
1.0
1.5
β_{IV}
